# Quantitative Analysis of Amorphous Form in Indomethacin by Near Infrared Spectroscopy Combined with Partial Least Squares Regression Analysis

**DOI:** 10.3390/molecules29225290

**Published:** 2024-11-08

**Authors:** Mingdi Liu, Rui Fu, Jichao Liu, Ping Song, Haichao Li, Weibing Dong, Zan Sun

**Affiliations:** 1 College of Chemistry and Materials Science, Qinghai Minzu University, Xining 810007, China; 2 Qinghai Provincial Key Laboratory of Nanomaterials and Technology, Qinghai Minzu University, Xining 810007, China; 3 Key Laboratory of Resource Chemistry and Eco-Environmental Protection in Tibetan Plateau, State Ethnic Affairs Commission, Xining 810007, China

**Keywords:** indomethacin, amorphous, quantitative analysis, NIR, PLSR

## Abstract

Indomethacin (INDO) is a synthetic non-steroidal antipyretic, analgesic, and anti-inflammatory drug that commonly exists in both amorphous and crystalline states. Its amorphous state (A-INDO) is utilized by pharmaceutical companies as an active pharmaceutical ingredient (API) in the production of INDO drugs due to its higher apparent solubility and bioavailability. The crystal state also encompasses various crystal forms such as the α-crystal form (α-INDO) and γ-crystal form (γ-INDO), with the highly crystalline and insoluble γ-INDO being commercially available. A-INDO, existing in a thermodynamically high-energy state, is susceptible to several factors during the preparation, storage, and transportation of API leading to its conversion into γ-INDO, thus impacting the bioavailability and efficacy of INDO drugs. Therefore, quantitative analysis of the A-INDO/γ-INDO content in INDO API becomes essential for controlling the production quality of INDO. The primary objective of this study is to investigate the feasibility of NIR for the quantitative analysis of A-INDO in INDO API, and to further elucidate its quantitative analysis mechanism. The NIR spectral data were collected for A-INDO and γ-INDO binary mixture samples with different resolutions, and these spectra were then selected and reconstructed using the interval partial least square (iPLS) method. Different pretreatment methods were employed to enhance the reconstructed spectra by highlighting relevant eigen information while eliminating invalid information caused by environmental factors or physical characteristics of samples. The most suitable PLSR model for quantitative analysis of A-INDO within the range of 0.0000–100.0000% *w*/*w*% was established, screened, and validated. From various perspectives, including distribution of spectral effective information, impact of resolution on PLSR model performance, variance contribution/cumulative variance contribution of PLSR model principal components (PCs), PC_I_ loadings, relationship between spectral scores, and A-INDO content, feasibility assessment was conducted for the quantitative analysis of A-INDO in INDO using NIR spectroscopy. Additionally, a detailed investigation on the quantitative analysis mechanism of the optimal PLSR model was undertaken including the correlation between the characteristic peaks of spectra and information regarding hydrogen groups or hydrogen bonds in A-INDO or γ-INDO molecules. This study aims to provide theoretical support for the quantitative analysis of A-INDO in INDO API as well as serve as a reliable reference method for API quantification and quality control in similar drugs.

## 1. Introduction

**Figure 1:**
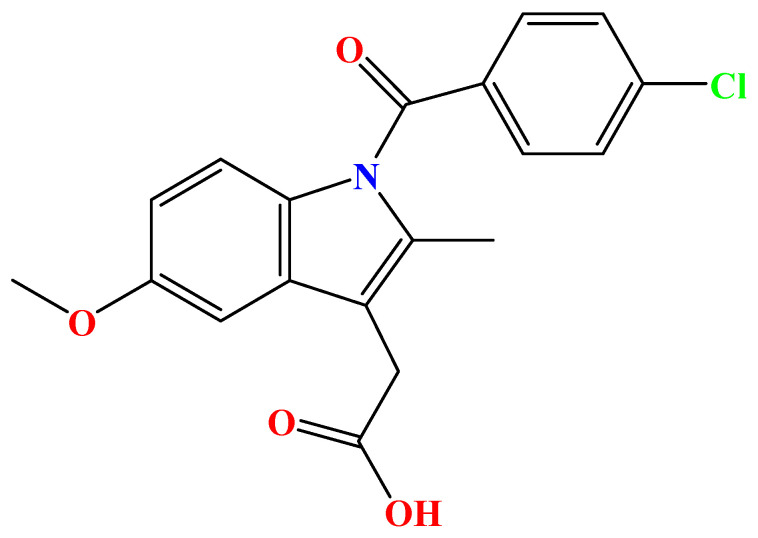
Chemical structure of indomethacin.

Active pharmaceutical ingredients (API) can exist in two different forms, namely the amorphous state and the crystalline state, depending on their packing arrangement [1,2]. The amorphous state represents a unique molecular arrangement of API [1]. In comparison to the long-range ordered crystalline state, the molecular arrangement of amorphous API exhibits characteristics of both long-range disorder and short-range order [1,2]. Amorphous API possesses similar rigidity to crystal API and isotropy similar to fluids, offering numerous advantages in applications that cannot be achieved by its crystalline counterpart [1,2]. Amorphous API is characterized by a high-energy disorder, which directly influences its drug properties, such as high solubility and fast dissolution rate [3,4,5,6]. Due to its significant benefits in enhancing solubility and bioavailability of BCS II drugs with low solubility and high permeability, it finds extensive usage in pharmaceutical preparations [5]. For example, indomethacin (C_19_H_16_ClNO_4_, 1-(4-chlorobenzoyl)-5-methoxy-2-methylindol-3-acetic acid), commonly known as INDO (Figure 1), is a synthetic non-steroidal antipyretic, analgesic, and anti-inflammatory drug. It encompasses various crystal forms such as the α-crystal form (α-INDO) and γ-crystal form (γ-INDO) [7,8,9], among which the commercially available γ-INDO with high crystallinity and insoluble γ-INDO are the typical crystal forms of INDO. However, its amorphous state (A-INDO) offers more significant advantages over γ-INDO in terms of solubility and bioavailability. Nevertheless, amorphous API is thermodynamically unstable and prone to spontaneous transformation into a lower-energy and more stable crystalline state during production/storage/transportation/processing [10,11]. Once transformed from an amorphous state to a crystalline state, it loses its high-solubility benefits, thereby affecting the quality of the final product. Therefore, improving evaluation methods for determining the content of crystal impurity in amorphous API during drug production and processing has become crucial for enhancing drug quality and clinical efficacy.

When the API undergoes a transition from an amorphous state to a crystalline state, significant transformations occur in its physical structure, energy state, thermodynamic properties, and optical properties [1,2,3,4]. Consequently, various techniques are available for qualitative analysis of amorphous API, including powder X-ray diffraction (PXRD) [12,13,14,15], near-infrared spectroscopy (NIR) [16,17], differential scanning calorimetry (DSC) [18,19] and Raman spectroscopy [19,20,21]. However, these techniques do not yield satisfactory results when it comes to quantitative analysis of amorphous API [22,23,24]. For example, collecting DSC maps of amorphous API may cause irreversible damage or induce a transition to a crystalline state. Fortunately, NIR exhibits sensitivity towards hydrogen bonds and hydrogen-containing groups (O-H, N-H, C-H) within API molecules, and has the advantages of convenience, speed, efficiency, accuracy, low cost, no sample destruction, no chemical reagent consumption, and no environmental pollution [25,26]. Henceforth it has been extensively employed in quantitative analysis of API polymorphism.

Although NIR possesses multiple advantages in API polymorphism quantitative analysis, it is susceptible to interference from external factors (such as electrical noise, stray light, background, etc.) during the process of sample data collection, resulting in the emergence of “noise”. Furthermore, the physical characteristics of the sample (including particle size, mixing uniformity, density, viscosity, surface finish) also exert an impact on the accuracy of spectral data. Therefore, selecting appropriate raw spectral data preprocessing and modeling methods is crucial for extracting effective information while discarding invalid and noisy information. This aids in enhancing distinguishable feature information (content information of each component) and establishing a model with excellent performance. Commonly employed NIR spectral data preprocessing techniques include Savitzky–Golay first derivative (SG1st) [21,27,28], Savitzky–Golay second derivative (SG2nd) [21,27,28], multiple scattering correction (MSC) [29,30], standard normal variable (SNV) [30,31], and wavelet transform (WT) [11,13,18,26,32]. Similarly, commonly utilized modeling methods encompass multiple linear regression (MLR) [33], principal component regression (PCR) [34,35], and partial least square regression (PLSR) [11,13,18,26,36,37], etc.

The primary focus of this study lies in the binary mixture of A-INDO and γ-INDO, aiming to establish quantitative analysis models for the content of A-INDO within the INDO binary mixture samples through the integration of NIR spectroscopy and PLSR, and trying to elucidate its feasibility and underlying quantitative analysis mechanism. The ultimate goal was to address the industry challenge of accurately detecting crystal impurities in amorphous API and thereby achieving impeccable quality control in pharmaceutical production.

## 2. Results and Discussion

### 2.1. Characterization of A-INDO and γ-INDO

**Figure 2:**
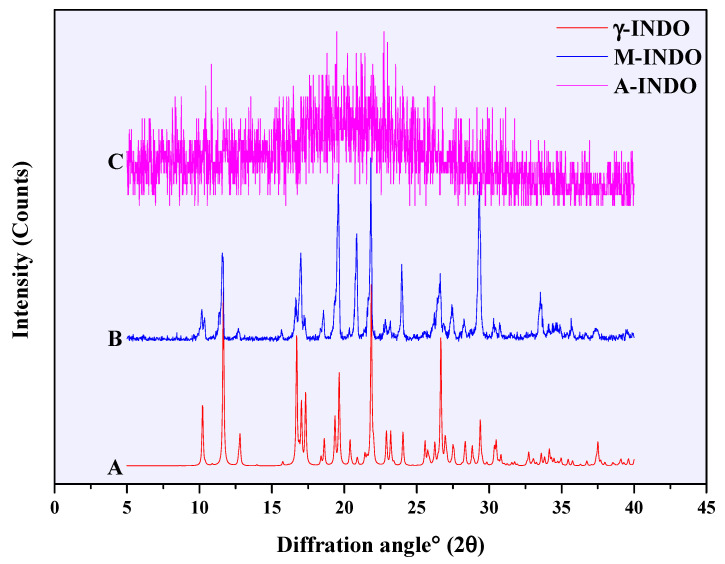
PXRD pattern of different INDO solid forms, (A) γ-INDO, (B) M-INDO, (C) A-INDO.

The PXRD patterns of INDO in different solid forms are presented in Figure 2. Curve B and curve A provide a comparison between the PXRD data of commercially available INDO (M-INDO) and γ-INDO obtained from the Cambridge Crystallographic Data Centre (CCDC). The peak positions of the characteristic peaks were found to be completely consistent, thereby confirming that the commercially available INDO was indeed γ-INDO. Curve C and curve B exhibit the PXRD data for prepared A-INDO and commercially available γ-INDO, respectively. It can be observed from the pattern that the characteristic peak of γ-INDO disappears, being replaced by an indicative amorphous halo, indicating successful preparation of A-INDO.

**Figure 3:**
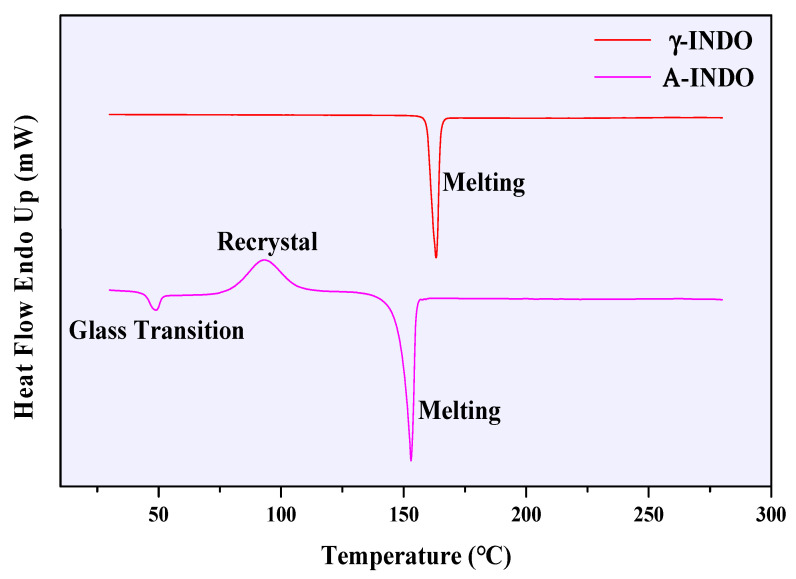
DCS curves of different INDO solid forms.

The DSC curves of γ-INDO and A-INDO are presented in Figure 3. In the DSC curve of γ-INDO, a single endothermic peak at 163 °C indicates melting. However, the DSC curve of A-INDO exhibits two distinct endothermic peaks corresponding to glass transition and melting, respectively, along with an exothermic peak indicating recrystallization as previously reported by Otsuka et al. [38]. These results confirmed the successful preparation of A-INDO.

**Figure 4:**
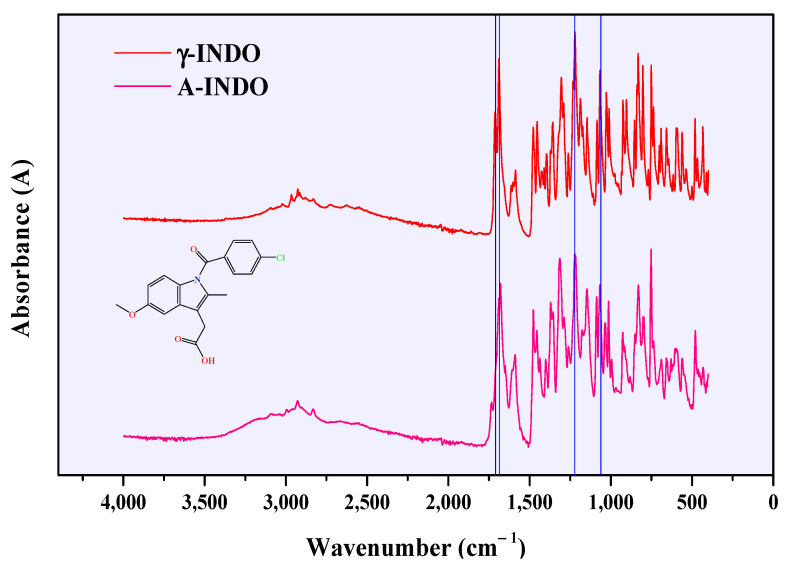
ATR-FTIR spectra of different INDO solid forms.

The ATR-FTIR spectra of different solid forms of INDO are shown in Figure 4, and the observed absorption peaks for γ-INDO and A-INDO align with those reported by Lynne and Zografi [39]. In Figure 4, a distinct stretching vibration absorption peak at 1712 cm^−1^ is observed for the carboxylic acid’s C=O bond, while another stretching vibration absorption peak at 1689 cm^−1^ manifests itself for the amide’s C=O bond. Additionally, two prominent absorption peaks corresponding to phenyl ether were detected at 1221 cm^−1^ and 1067 cm^−1^.

**Figure 5:**
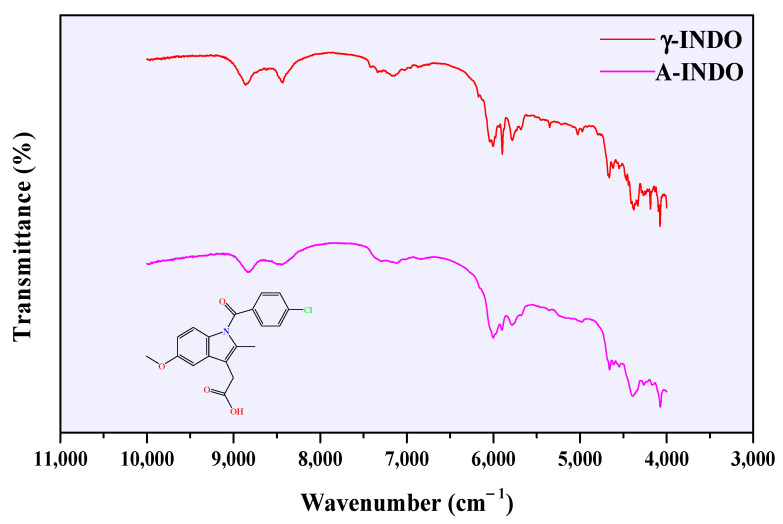
NIR spectra of different INDO solid forms.

NIR spectra of different solid forms of INDO are illustrated in Figure 5. According to Otsuka et al. [38], the distinctive peaks at wavenumbers of 8532 cm^−1^, 8432 cm^−1^, 5940 cm^−1^, and 4656 cm^−1^ are the presentation of functional groups -CH_3_, HC=CH, aromatic ring, and HC=CH in INDO molecules, respectively.

### 2.2. Construction of NIR Spectral Database

**Figure 6:**
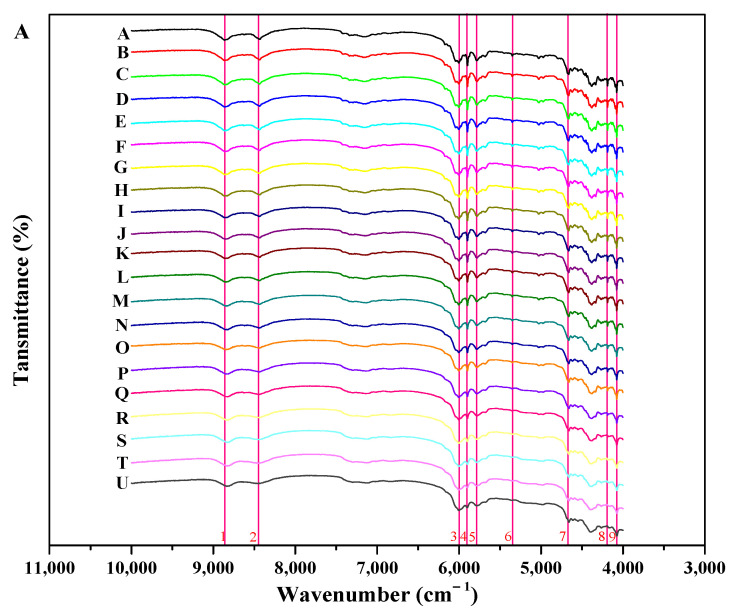

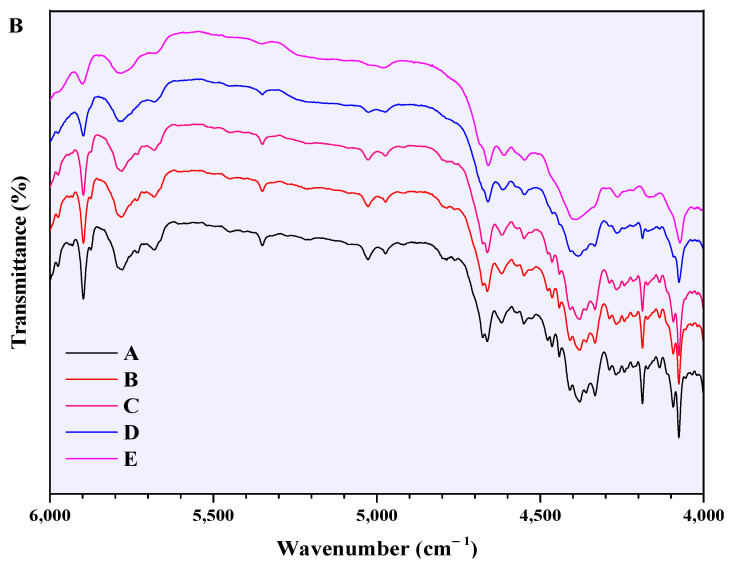
NIR spectra of binary mixture samples of INDO. (**A**,**B**) are the NIR spectra at 10,000–4000 cm^−1^ and 6000–4000 cm^−1^ of binary mixture samples (2 cm^−1^), respectively.

Different-resolution NIR spectra of 21 binary mixtures samples with different A-INDO contents are shown in Figure S1 for quantification of A-INDO. NIR spectra of 21 binary mixture samples with different A-INDO contents and resolution of 2 cm^−1^ are illustrated in Figure 6. In Figure 6A, nine regions which exhibit significant alterations in peak shapes are identified by pink lines. Simultaneously, an obvious trend indicating a reduction in transmission peak intensity with increasing A-INDO content is observed in Figure 6B, providing an opportunity for quantitative analysis of A-INDO using NIR spectroscopy. However, it should be noted that the decreasing trend of individual transmission peak intensity was not linear as the A-INDO content increased, suggesting that establishing an accurate quantitative analysis method based on the relationship between individual peak intensity/peak area and A-INDO content may pose challenges.

### 2.3. Establishment of PLSR Models

**Figure 7:**
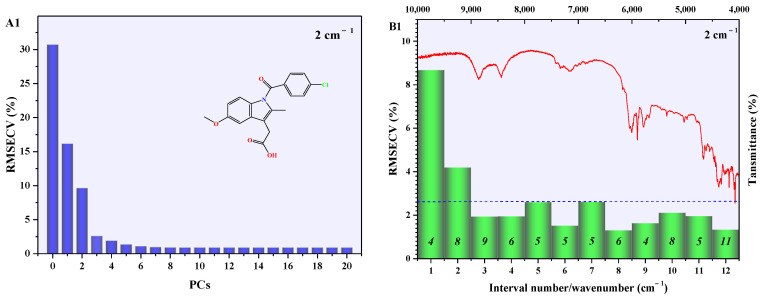

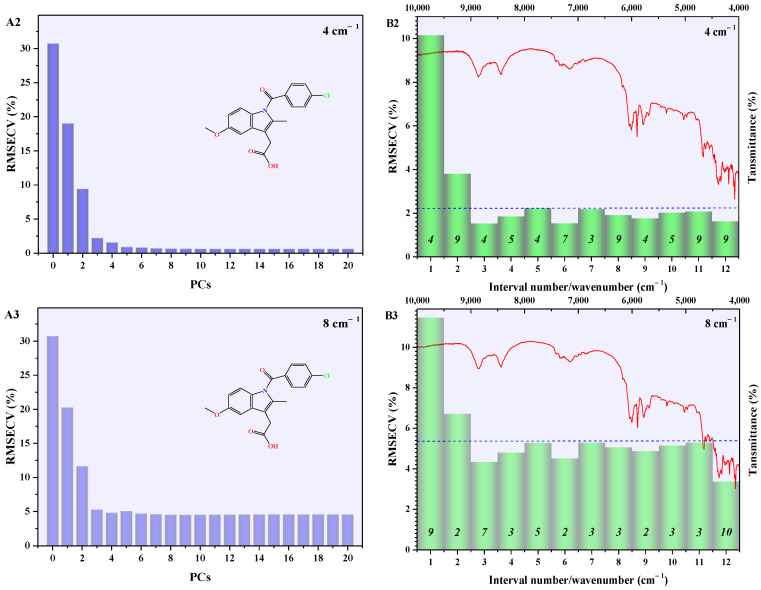
The RMSECV of PLSR models. (**A1**–**A3**) are the RMSECV vs. components of global PLSR models with different resolutions; (**B1**–**B3**) are the RMSECV of IPLS interval models with different resolutions, while the dotted line is the RMSECV for the global PLSR model. Italic numbers are optimal principal components of interval models.

NIR data (10,000–4000 cm^−1^) of INDO binary mixture samples with different resolutions (2 cm^−1^, 4 cm^−1^, 8 cm^−1^) contained a significant amount of redundant information, resulting in prolonged model analysis time and increased difficulty in model analysis. Therefore, the original NIR data were divided into 12 equal-width subintervals according to wavenumber. Subsequently, PLSR models for quantitative analysis of A-INDO content in INDO binary mixture samples were established using both the full spectrum and each subinterval, separately. RMSECV of the PLSR models for the full spectrum and subinterval were calculated using cross-validation on the validation set. Furthermore, with RMSECV as the standard, a PLSR model for predicting A-INDO content was developed by selecting corresponding bands from the subinterval as calibration set data. RMSECV diagrams (Figure 7(A1–A3)) of full-spectrum PLSR models with different numbers of principal components (PCs) and different resolutions (2 cm^−1^, 4 cm^−1^, and 8 cm^−1^) show that when PCs numbers (N) are around three, they yield the RMSECV < 5.0000%. Therefore, the RMSECV of the full-spectrum PLSR models with 3 PCs was used as an indicator to investigate the subinterval. The RMSECV value and number of PCs for the PLSR model with the optimal subinterval are shown in Figure 7(B1–B3).

**Figure 8:**
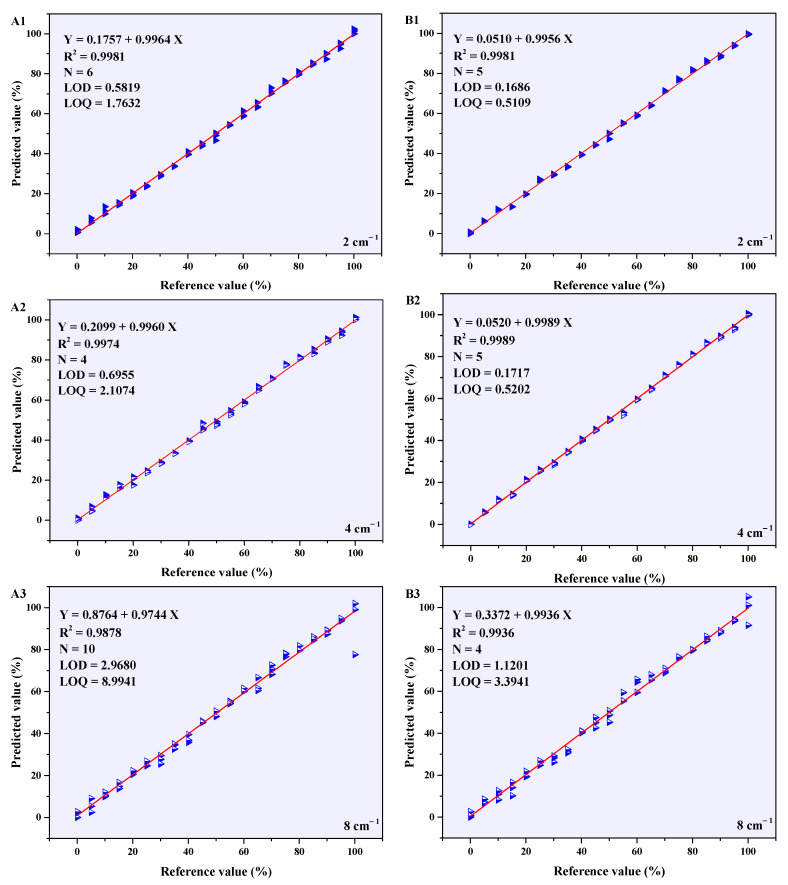
PLSR model calibration curves for NIR data with different resolutions. (**A1**–**A3**) are optimal iPLS model calibration curves of NIR data with different resolutions, (**B1**–**B3**) are PLSR model calibration curves of NIR data with different resolutions of 9000–4000 cm^−1^.

Different-resolution subinterval PLSR model RMSECV values that were lower than the full-spectrum PLSR model RMSECV values’ corresponding subinterval sequence numbers were 3–12, which represented the wavenumber range from 9000 cm^−1^ to 4000 cm^−1^. These results indicate that the crucial information for quantitatively analyzing A-INDO content in the NIR spectra of a binary mixture is primarily concentrated within the region of 9000–4000 cm^−1^. When the resolution was 2 cm^−1^, the RMSECV value of the PLSR model in subinterval 8 (6500–6000 cm^−1^) was the smallest, indicating that the PLSR model established in this subinterval had the best performance. The calibration curve is shown in Figure 8(A1), with LOD = 0.5819% and LOQ = 1.7632%. When the resolution was 4 cm^−1^, the minimum RMSECV value of PLSR model occurred at subinterval 3 (9000–8500 cm^−1^), and the calibration curve in Figure 8(A2) shows the LOD = 0.6955% and LOQ = 2.1074%. When the resolution was 8 cm^−1^, a similar situation appeared in subinterval 12 (4500–4000 cm^−1^). The calibration curve in Figure 8(A3) shows the LOD = 2.9680% and LOQ = 8.9941%.

The *R*^2^ of optimal iPLS models with different resolutions, shown in Figure 8(A1–A3), could exceed 0.9800. Among them, the resolution of 2 cm^−1^ and 4 cm^−1^ performed better and could be used for quantitative analysis of A-INDO content in INDO binary mixtures. However, the LOD and LOQ were relatively high, making it unsuitable for analyzing low-content A-INDO. To optimize the PLSR model, NIR spectral data (9000–4000 cm^−1^) were reconstructed using all the subinterval spectral data with a smaller RMSECV value compared to that of full-spectrum PLSR models. Various pretreatment methods were employed to preprocess the reconstructed data, and then PLSR models were established. The optimal PLSR models and their parameters for reconstructed data with resolution of 2 cm^−1^, 4 cm^−1^, and 8 cm^−1^ are presented in Table 1. Additionally, Figure 8(B1–B3) illustrates the calibration curves of these models.

The comparison of the parameters of the optimal PLSR models for reconstructed NIR data with different resolutions revealed that as the resolution increased, the number of PCs decreased. This suggests that higher resolution confers an advantage in discerning component-related information in the INDO binary mixtures. The *R*^2^ exhibited an initial increase followed by a decrease, indicating that improved resolution aids in distinguishing information about each component in the binary mixtures, while also enhancing noise resolution. RMSECP and RMSEP initially decreased and then increased, suggesting that enhancing resolution contributes to better discrimination among components in the binary mixture. However, excessively high resolution should be avoided. LOD and LOQ values of the PLSR model are comparable at 2 cm^−1^ and 4 cm^−1^ resolutions but become larger at 8 cm^−1^, which impedes quantitative analysis when A-INDO content falls below 3.3941%.

The full-spectrum PLSR models with resolution of 4 cm^−1^ exhibited the lowest RMSECV values compared to those with resolution of 2 cm^−1^ and 8 cm^−1^ (as depicted in Figure 7(A1–A3)). Additionally, the RMSECV values of the 12 iPLS models with a resolution of 4 cm^−1^ were minimized compared to those with a resolution of 2 cm^−1^ and 8 cm^−1^ (shown in Figure 7(B1–B3)). Therefore, it is considered that the PLSR model of reconstructed NIR data (9000–4000 cm^−1^) with a resolution of 4 cm^−1^ established after SNV + WT pretreating is the most suitable model for quantitative analysis of A-INDO content in INDO binary mixture samples, and exhibiting minimal prediction error for unknown A-INDO levels. It also demonstrates capability in detecting and quantifying low-content of A-INDO.

### 2.4. Verification and Evaluation of the Optimal Model for Reconstructed NIR Data

The optimized PLSR models were validated by reformulating and repreparing a series of INDO binary mixture samples with known A-INDO content (5.0000%, 15.0000%, 25.0000%, 35.0000%, 45.0000%, and 70.0000%). The NIR data were collected and subjected to identical pretreatment methods, followed by utilizing the established PLSR model to predict the A-INDO content of these known samples, thereby assessing the performance of the developed model in terms of prediction accuracy. Additionally, specific attention was given to collecting corresponding data from a sample containing A-INDO content at 45.0000% for methodological investigation purposes. The detailed results are presented in Table 2.

The different-resolution optimal PLSR models were established using reconstructed NIR data, which successfully predicted the content of A-INDO in six known samples. These prediction results showcased an exceptional level of accuracy since they closely aligned with the reference values. Furthermore, the disparities between the predicted values and reference values were minimal, signifying outstanding predictive performance. When comparing prediction errors among PLSR models with different resolutions for A-INDO content ranging from 0.0000% to 100.0000% *w*/*w*%, it was observed that higher-resolution models yielded superior overall predictions. However, it is noteworthy that both the 2 cm^−1^ and 8 cm^−1^ resolutions exhibited slightly larger prediction errors compared to the more precise 4 cm^−1^ resolution model. Consequently, it can be concluded that employing a PLSR model established by reconstructed NIR data at a resolution of 4 cm^−1^ offers unparalleled performance in quantitatively analyzing A-INDO content.

### 2.5. Discussion on Quantitative Mechanism of PLSR Models

**Figure 9:**
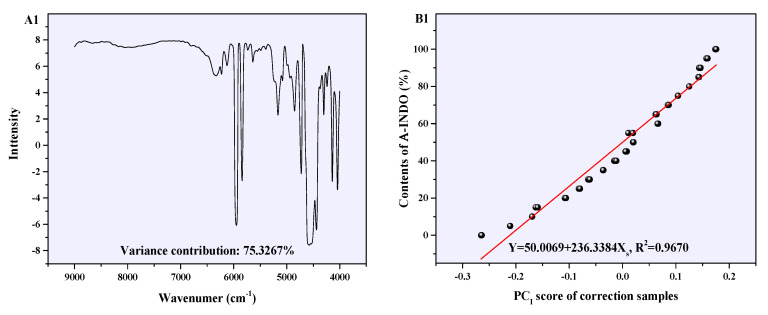

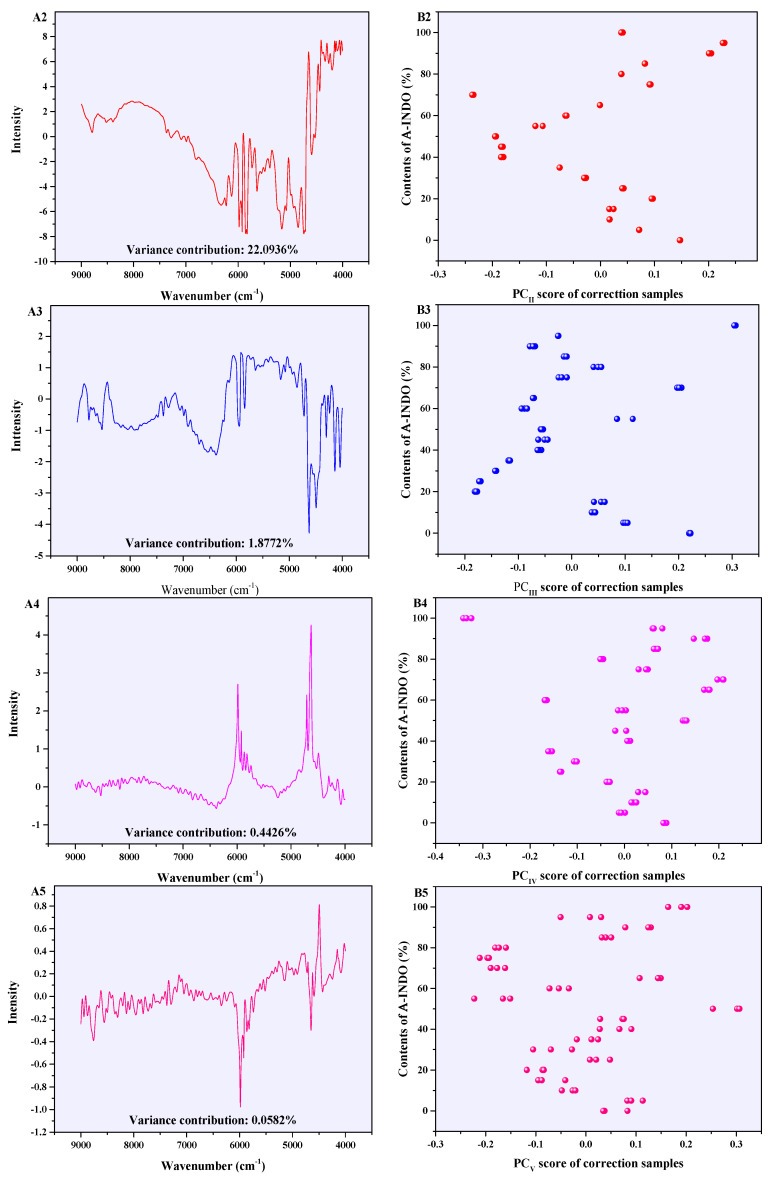
The loadings (**A1**–**A5**) and the scores (**B1**–**B5**) of the PLSR model established after pretreated SNV + WT in 9000–4000 cm^−1^ with resolution 4 cm^−1^.

PLSR model of the reconstructed NIR spectral data (9000–4000 cm^−1^) with resolution of 4 cm^−1^, mentioned earlier, was found to be the most suitable for quantitative analysis of the content of A-INDO. Further discussion on this model is presented here. The five PCs of this model (accumulative variance contribution was 99.7983%) loadings and the relation between PC scores are shown in Figure 9. PC_I_, as shown in Figure 9(A1), explained 75.3267% of the sample information, and its loadings diagram was consistent with the reconstructed NIR spectral of A-INDO with pretreated by SNV + WT, as shown in Figure S2. This indicated that PC_I_ mainly expressed the information of A-INDO in binary mixtures. Meanwhile, a relatively perfect quantitative relation between PC_I_ score and A-INDO content was obtained, which can be used as the calibration curve for quantitatively predicting the A-INDO content using the spectra. The relation between its score and A-INDO content was Y = 50.0069 + 236.3384 X_S_, *R*^2^ = 0.9670 (as shown in Figure 8(B1)). The PC_II_ explained 22.0936% (as shown in Figure 9(A2)) of the sample information and its loadings diagram was consistent with the reconstructed NIR spectrum of γ-INDO pretreated by SNV + WT, as shown in Figure S3, suggesting that PC_II_ mainly explained information associated with γ-INDO in binary mixtures. Although the sample information of PC_III_ (1.8772%, as shown in Figure 9(A3)), PC_IV_ (0.4426%, as shown in Figure 9(A4)), and PC_V_ (0.0582%, as shown in Figure 9(A5)) was less, they made important contributions to improve the model performance. These five PCs of this model covered 99.7983% information in the NIR spectrum of the binary mixture, indicating that the utilized effective information closely approximated the reconstructed raw NIR spectrum data. Therefore, it could be concluded that this model was reliable for quantitatively analyzing A-INDO content in the binary mixture.

**Figure 10:**
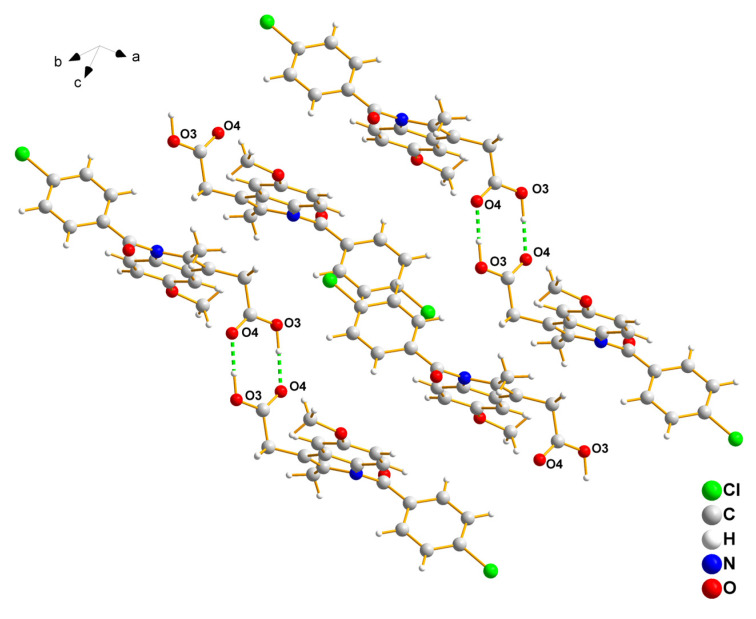
Hydrogen bonds in γ-INDO.

The γ-INDO crystal system belongs to the triclinic class of space group $P\bar{1}$. A dimer structure is formed through the hydrogen bonding of the inter-molecular carboxylic acid groups near the inversion center, and an inter-molecular C-H···π hydrogen bond is also observed, as illustrated in Figure 10. The A-INDO represents an amorphous solid substance that lacks a periodic arrangement, and INDO molecules are disordered in three-dimensional space, resulting in weakened or absent intermolecular hydrogen bonds.

According to Workman et al. [40], in the NIR spectrum of A-INDO, a combined frequency at 4659 cm^−1^ signifies the stretching vibration of carboxylic acid group OH and CO bonds, while another combined frequency at 4611 cm^−1^ corresponds to the stretching vibrations of C-H bonds and C=O bonds. Additionally, a combined frequency at 4166 cm^−1^ indicates the stretching vibration of C-H bonds along with the bending vibration of COH groups, whereas a combined frequency at 4073 cm^−1^ suggests the stretching vibrations of C-H bonds and CO bonds. However, the γ-INDO exhibits a dimeric structure attributed to the formation of hydrogen bonds between carboxylic acid groups. The combined frequencies of the stretching vibrations of OH and CO for the carboxylic acid group are observed at 4662 cm^−1^ and 4658 cm^−1^, respectively. The combined frequency of C-H stretching vibration and C=O stretching vibration is detected at 4618 cm^−1^. Additionally, the combined frequency of OH stretching vibration and OH-O bending vibration occurs at 4528 cm^−1^. Furthermore, the combined frequencies of OH stretching vibration and CH bending vibration split into two peaks: one at 4468 cm^−1^ and another at 4464 cm^−1^, respectively. Moreover, the combined frequency of C-H stretching vibration and OH-O bending vibration appears at 4333 cm^−1^, while that of C-H stretching vibration and COH bending vibrations is observed at 4188 cm^−1^. Finally, the combined frequencies of C-H stretching vibrations and CO stretching vibrations split into two peaks: one at 4093 cm^−1^ and another at 4076 cm^−1^.

The hydrogen bond and its corresponding transmission peaks gradually diminish with decreasing A-INDO content in the INDO binary mixture until they completely disappear, as illustrated in Figure 6B. The bands selected by iPLS for establishing PLSR models contain only these hydrogen bonds and corresponding transmission peaks influenced by them, indicating that the NIR spectrometric quantitative analysis of A-INDO content in INDO binary mixtures relies on the hydrogen bonds in γ-INDO molecules and the changes of corresponding transmission peaks influenced by hydrogen bonds.

## 3. Materials and Methods

### 3.1. Materials and Samples Preparation

#### 3.1.1. Materials

The γ-INDO compound (purity > 99.9%, melting point of γ-INDO = 163 °C, molecular weight = 357.79 g·mol^−1^) was procured from Dalian Meilun Biotechnology Co., Ltd. (Dalian, China). A-INDO was obtained by melting an appropriate amount of γ-INDO in an aluminum vessel at a temperature of 165 °C within a preheated oven for 30 min. Subsequently, the aluminum vessel was promptly removed and the fully molten γ-INDO compound was transferred into a dry and sterile beaker utilizing liquid nitrogen as a cryogenic coolant agent during this process step. Upon attaining ambient conditions, the specimen underwent collection followed by storage within a desiccator containing phosphorus pentoxide maintained at 4 °C until subsequent analytical investigations could be performed accordingly thereafter.

Both solid forms of INDO were subjected to PXRD and DSC characterization techniques to ascertain their respective polymorphic purities, with these two distinct solid phases serving as reference standards for quantitative analyses.

#### 3.1.2. Preparation of the Binary Mixture Samples

**Figure 11:**
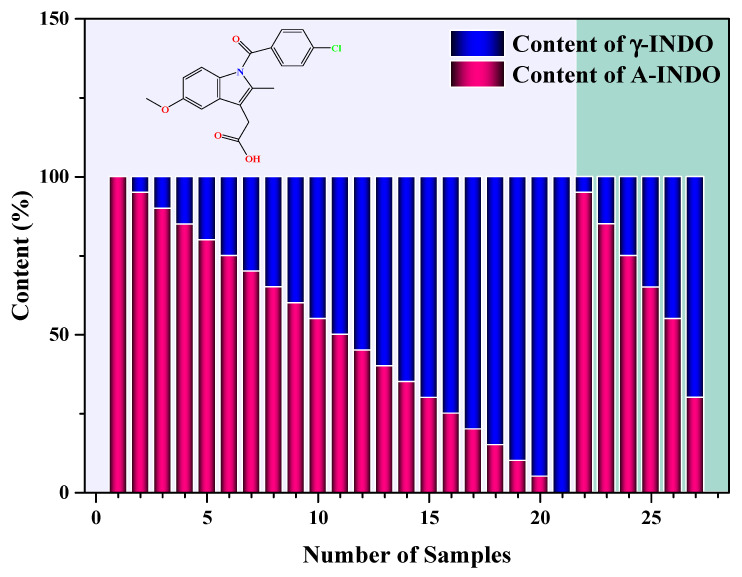
Samples used to establish and verify the quantitative models. Samples contained A-INDO and γ-INDO content of A-INDO were 0.0000, 4.9955, 10.0150, 15.0415, 20.0200, 25.0050, 30.0080, 34.9760, 40.0440, 45.0255, 50.0100, 54.9915, 59.9740, 65.0340, 70.0360, 74.9825, 79.9860, 85.0230, 89.9660, 95.0105,100.0000, 5.0000, 15.0005, 25.0000, 34.9995, 45.0020, and 70.0030 *w*/*w*%, respectively.

The A-INDO and γ-INDO were finely ground using an agate mortar before being passed through 100 mesh sieves to achieve particle size uniformity. Precise weighing was performed on a Mettler Toledo electronic balance (ME204T/02, Mettler Toledo Instruments Ltd., Shanghai, China), as outlined in Figure 11, to prepare binary mixture samples for quantitative analysis. To ensure the homogeneity and consistency of powder particles, each sample was subjected to grinding in an agate mortar for 5 min followed by thorough mixing in a laboratory vortex mixer for 20 min prior to collecting NIR data. The grinding and mixing experiments were carefully monitored using PXRD techniques to prevent any occurrence of phase transition.

### 3.2. Methods

The different solid forms of INDO powder were characterized using techniques including PXRD, DSC, ATR-FTI, and NIR.

#### 3.2.1. Powder X-Ray Diffraction

The PXRD patterns were acquired using a Rigaku R-AXIS-RAPID powder diffractometer (Rigaku Co., Akishima, Japan) equipped with Cu Ka radiation (k = 1.5405 A°). The instrument operated at an optimal ambient condition, with an operating voltage of 40 kV and current of 100 mA. The data were collected over a 2θ range of 2–50° with a step size of 0.02° and a scan rate of 8°·min^−1^. All samples underwent three meticulous rounds of PXRD analysis.

#### 3.2.2. Differential Scanning Calorimetry

The DSC measurements were performed using a Mettler DSC system (DSC, DSC 1/500, Mettler Toledo, Greifensee, Switzerland) with a precisely controlled nitrogen flow rate of 50 mL·min^−1^. Each sample weighing approximately 10 mg was subjected to heating from 30 °C to 280 °C at a carefully regulated rate of 10 °C·min^−1^ and tested in triplicate.

#### 3.2.3. Attenuated Total Reflectance Fourier-Transform Infrared Spectroscopy

The ATR-FTIR spectral data were acquired using a Bruker Alpha ATR-FTIR instrument (Billerica, MA, USA), which operated within an impressive spectral range of 4000–400 cm^−1^. The instrument exhibited a remarkable resolution of 4 cm^−1^, with a wavenumber interval of 1 cm^−1^ and cumulative scanning was performed 16 times. Each sample was scanned in triplicate.

#### 3.2.4. Near-Infrared Spectroscopy

The NIR spectra were analyzed using a near-infrared spectrometer (Thermo Fisher Scientific Antaris II, Waltham, MA, USA) with a resolution of 4 cm^−1^ and a wavenumber interval of 1 cm^−1^, covering the range from 10,000 cm^−1^ to 4000 cm^−1^. Cumulative scans were performed for each sample a total of 16 times. To ensure accuracy, three consecutive scans were conducted for each sample to obtain an average value. This meticulous process was repeated twice, resulting in the generation of three sets of meticulously averaged spectral data for further analysis.

### 3.3. Pretreatment of Spectra Data

A total of 63 NIR spectra (comprising three average spectral data for each sample) were acquired to develop and validate quantitative models for the determination of A-INDO content in binary mixtures. The interval partial least squares (iPLS) method, along with various preprocessing techniques, was employed to mitigate the influence of spurious information caused by scattering, variations in light path due to uneven particle distribution, and different particle sizes on the NIR data. These techniques effectively aligned the spectral data of processed samples with their intrinsic information. Subsequently, PLSR analysis was performed using MATLAB2014a to establish robust PLSR models for NIR analysis. Linear regression data processing was conducted using Origin software (OriginPro 9.1 64Bit). The model’s performance was assessed based on correlation coefficient (*R*^2^), root mean square error of calibration (RMSEC), cross-validation (RMSECV) values, and prediction error (RMSEP) values. These evaluation metrics were calculated according to Equations (1) and (2),

(1) $$R^{2}=1-\left(\sum_{i=1}^{n}{\left(y_{i}-{\hat{y}}_{i}\right)}^{2}/\sum_{i=1}^{n}{\left(y_{i}-\bar{y}\right)}^{2}\right)$$

(2) $$RSME=\sqrt{\sum_{i=1}^{n}{\left(y_{i}-{\hat{y}}_{i}\right)}^{2}/n}$$

where $y_{i}$, ${\hat{y}}_{i}$, $\bar{y}$, and n in the Equations (1) and (2) represented theoretical value, calculated value, average value, and the number of samples, respectively.

### 3.4. Validation of Developed Models

To validate the accuracy of the correction models, NIR techniques were employed to scan samples with known A-INDO content (5.000%, 15.000%, 25.000%, 35.000%, 45.000%, and 70.000%), and subsequently, the established PLSR model was utilized for prediction purposes. Simultaneously, the precision, repeatability, and stability of NIR techniques were assessed using A-INDO content at a concentration level of 45.000%. The relative standard deviation (RSD%) was calculated for each PLSR model according to Equation (3), while the limit of detection (LOD) and limit of quantification (LOQ) were determined for each PLSR model utilizing Equations (4) and (5),

(3) $$RSD=\left(\sqrt{\sum_{i=1}^{n}{\left({\hat{y}}_{i}-\bar{y}\right)}^{2}/n-1}/\bar{y}\right)\times 100\%$$

(4) LOD=3.3σ/s

(5) LOQ=10σ/s

where ${\hat{y}}_{i}$, $\bar{y}$, n, σ, s in Equations (3)–(5) were predicted content values, average of the predicted content values of the samples, number of the samples, standard deviation of predicted content values, and slope of the calibration curve, respectively.

## 4. Conclusions

The aim of this study was to investigate the feasibility and principles of NIR spectroscopy for quantitatively analyzing the A-INDO/γ-INDO content in INDO binary mixtures, as well as to establish a rapid, accurate, non-destructive, and non-contact quantitative analysis method. This would enable better control over the production quality of INDO drugs. NIR was used to collect spectral data from A-INDO and γ-INDO binary mixture samples with different resolutions (2 cm^−1^, 4 cm^−1^, and 8 cm^−1^). The original NIR spectral data were then selected using the iPLS method and reconstructed. Various preprocessing techniques were applied to highlight the relevant information related to A-INDO and γ-INDO in the reconstructed spectra while eliminating any invalid information caused by environmental factors or physical characteristics of the samples. Finally, a suitable PLSR model for quantitative analysis of A-INDO within the range of 0.0000~100.0000% *w*/*w*% was established and validated.

By comparing the PLSR models of 12 subintervals divided using the iPLS method, it has been observed that the pertinent information for A-INDO quantitative analysis is predominantly concentrated within the wavenumber range of 9000–4000 cm^−1^, which reflects both fundamental frequency vibration overtones and combined information related to hydroxyl and hydrogen bonds. Furthermore, when comparing the optimal PLSR models of reconstructed spectral data (9000~4000 cm^−1^) with different resolutions, it has been noticed that enhancing the resolution of NIR spectroscopy enables clear differentiation between A-INDO and γ-INDO in the INDO binary mixtures. However, simultaneously improving resolution also enhances the irrelevant information. Therefore, the PLSR model established after SNV + WT preprocessing of reconstructed NIR spectral data with a resolution of 4 cm^−1^ was selected as the quantitative analysis model for A-INDO in the binary INDO mixtures. The model is Y = 0.0510 + 0.9956 X, with *R*^2^ = 0.9981, N = 5, and its LOD and LOQ are 0.1686 and 0.5109% *w*/*w*%, respectively. The feasibility and reliability of quantitative analysis of A-INDO in INDO binary mixtures by NIR spectroscopy were further elucidated based on the number of PCs, the variance contribution of PC_I_, the cumulative variance contribution of PCs, and the relationship between PC_I_ score and the content of A-INDO in this PLSR model. Finally, the quantitative analysis mechanism of the PLSR model was further elucidated by establishing a correlation between the NIR spectral characteristic peaks and A-INDO or γ-INDO.

The study can provide theoretical support for the quantitative analysis of A-INDO in INDO API as well as serve as a reliable reference method for API quantification (such as quantitative analysis of low-content impurity crystal forms in canagliflozin tablets [26]) and quality control in other drugs.

## Figures and Tables

**Table 1:** Optimal PLSR models for reconstruction NIR data with different resolutions.

Pretreatment Methods	Resolution	N	CVV ^a^	RMSECV (%)	RMSECP (%)	RMSEC (%)	*R* ^2^	LOD	LOQ
MSC + WT	2 cm^−1^	5	−0.1257	0.0021	1.9474	1.3074	0.9981	0.1686	0.5109
SNV + WT	4 cm^−1^	5	0.0841	0.0027	0.6263	1.0169	0.9989	0.1717	0.5202
SG1st + WT	8 cm^−1^	4	−0.3696	0.0067	3.4736	2.4271	0.9936	1.1201	3.3941

^a^ CVV shows the parameters of cross-validation validity.

**Table 2:** Validation results of the optimal PLSR models for reconstructed NIR data.

Samples	Reference Values (%)	Predicted Values		
		MSC + WT (2 cm^−1^)	SNV + WT (4 cm^−1^)	SG^1st^ + WT (8 cm^−1^)
V1 ^a^	5.0000	8.3832	5.6911	7.8797
V2 ^a^	15.0000	13.2967	13.8727	13.7179
V3 ^a^	25.0000	27.2036	25.5718	27.1908
V4 ^a^	35.0000	33.4986	34.6379	28.1575
V5 ^a^	45.0000	44.3445	45.3479	48.1177
V6 ^a^	70.0000	70.9312	70.1603	68.9421
Precision (RSD%)	45.0000	5.1493	3.3828	4.3653
Repeatability (RSD%)	45.0000	5.0216	3.1847	5.6319
Stability (RSD%)	45.0000	3.7537	3.1162	3.6846

^a^ V1, V2, V3, V4, and V5 were the samples used to validate quantitative models.

## Data Availability

Data are contained within the article and Supplementary Materials.

## Supplementary Materials

The following supporting information can be downloaded at: https://www.mdpi.com/article/10.3390/molecules29225290/s1, Figure S1: NIR spectra with different resolution of γ-INDO and A-INDO binary mixtures samples; Figure S2: Comparison between PCI loadings and reconstructed NIR spectra (after SNV + WT preprocessing) A-INDO; Figure S3: Comparison between PCI loadings and reconstructed NIR spectra (after SNV + WT preprocessing) A-INDO.
