# Supplementary material for: Quantitative Analysis of Amorphous Form in Indomethacin by Near Infrared Spectroscopy Combined with Partial Least Squares Regression Analysis
Source: Molecules. 2024 Nov 8;29(22):5290. doi: 10.3390/molecules29225290 (PMC11596882; doi:10.3390/molecules29225290)
Supplement: Supplementary file 1 [file molecules-29-05290-s001.zip › molecules-3238435-supplementary.pdf]

Quantitative Analysis of Amorphous Form in Indomethacin by Near Infrared Spectroscopy Combined with Partial Least Squares Regression Analysis

Mingdi Liu [1,2,3,*], Rui Fu [1,3], Jichao Liu [1,3], Ping Song [1,2,3], Haichao Li [1,2,3], Weibing Dong [1,2,3] and Zan Sun [1,2,3]

[1] College of Chemistry and Materials Science, Qinghai Minzu University, Xining 810007, China
[2] Qinghai Provincial Key Laborary of Nanomaterials and Technology, Qinghai Minzu University, Xining 810007, China
[3] Key Laboratory of Resource Chemistry and Eco-Environmental Protection in Tibetan Plateau, State Ethnic Affairs Commission, Xining 810007, China
* Correspondence: liumingdi@tju.edu.cn; Tel.: +86-971-8173864

SUPPLEMENTARY INFORMATION

NIR spectra with different resolution of γ-INDO and A-INDO binary mixtures samples were shown in Fig. S1.

Comparison between PC_{I} loadings and reconstructed NIR spectra (after SNV+WT preprocessing) A-INDO was shown in Fig. S2.

Comparison between PC_{II} loadings and reconstructed NIR spectra (after SNV+WT preprocessing) A-INDO was given in Fig. S3.

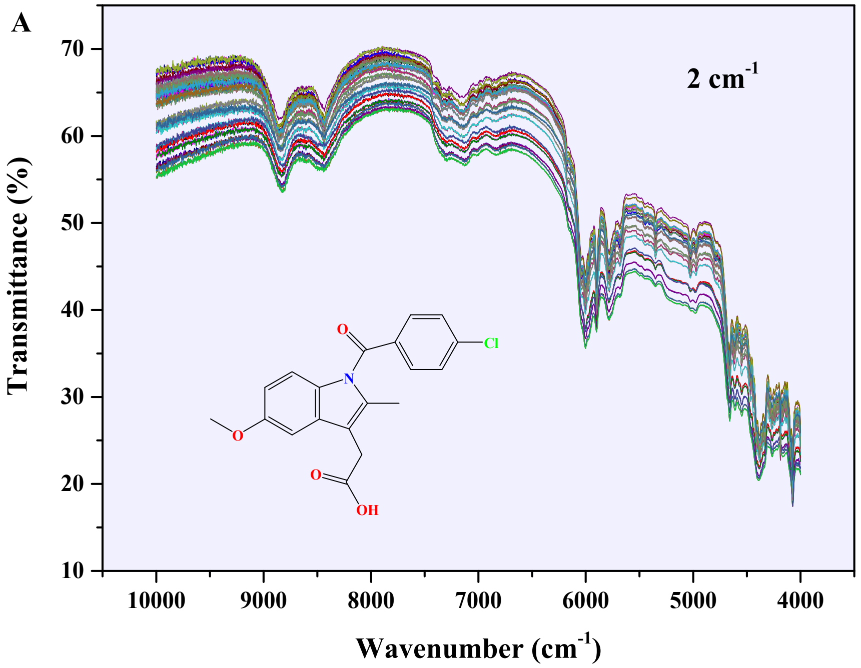
A
2 cm-1
Transmittance (%)
70
60
50
40
30
20
10
10000
9000
8000
7000
6000
5000
4000
Wavenumber (cm-1)
O
N
Cl
O
O
OH

(A)

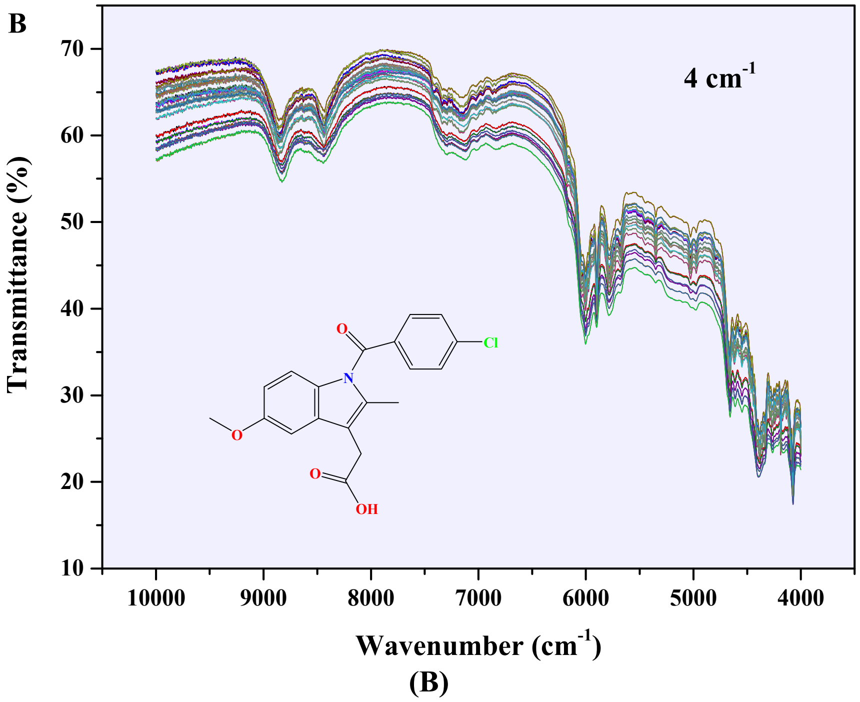
B
4 cm-1
Transmittance (%)
70
60
50
40
30
20
10
10000
9000
8000
7000
6000
5000
4000
Wavenumber (cm-1)
O
N
Cl
O
O
OH

(B)

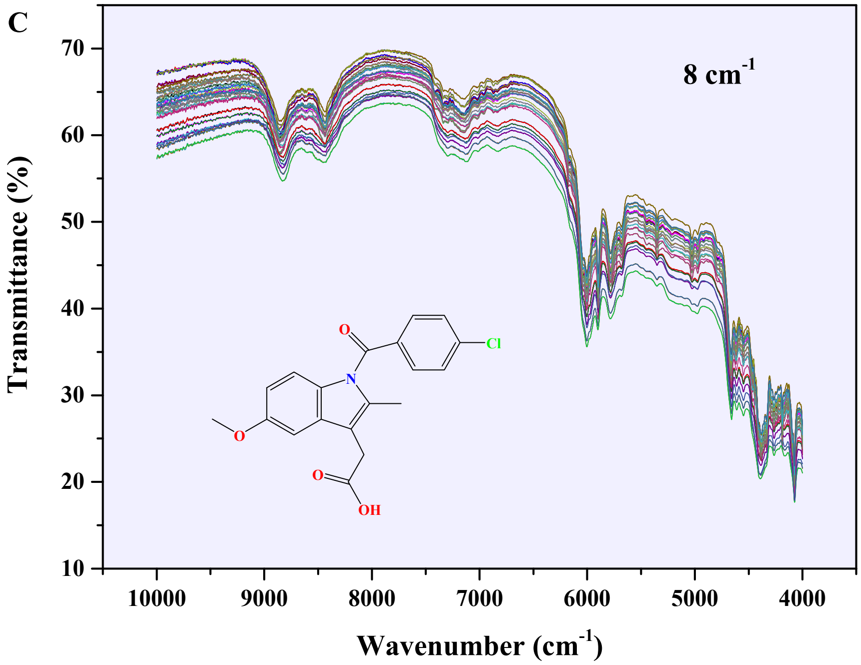

(C)

Figure. S1. NIR spectra with different resolution of γ-INDO and A-INDO binary mixtures samples. A, B and C were NIR spectra with a resolution of 2 cm^{-1}, 4 cm^{-1} and 8 cm^{-1}, respectively.

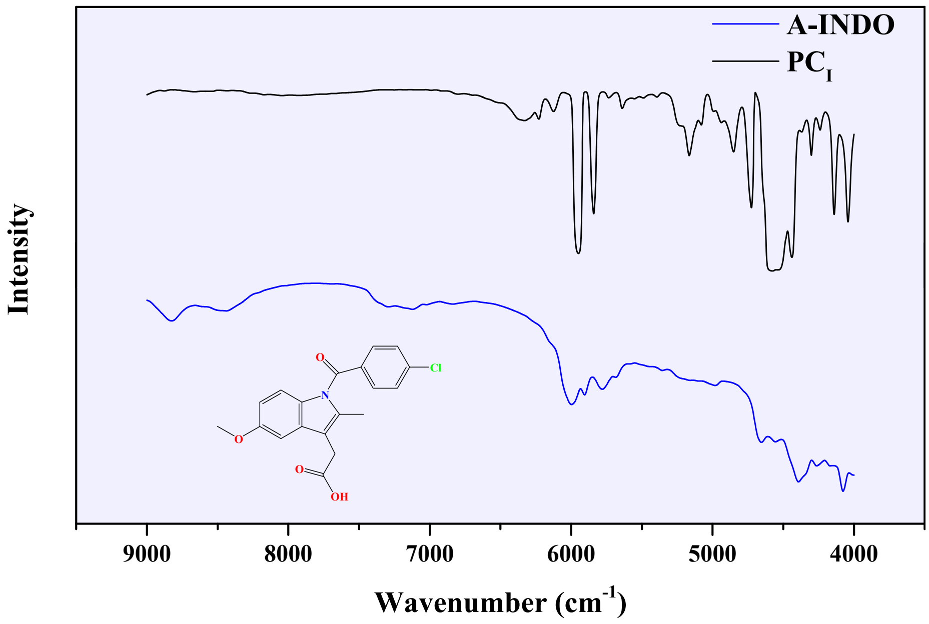

Figure. S2 Comparison between PC_I loadings and reconstructed NIR spectra (after SNV+WT preprocessing) A-INDO.

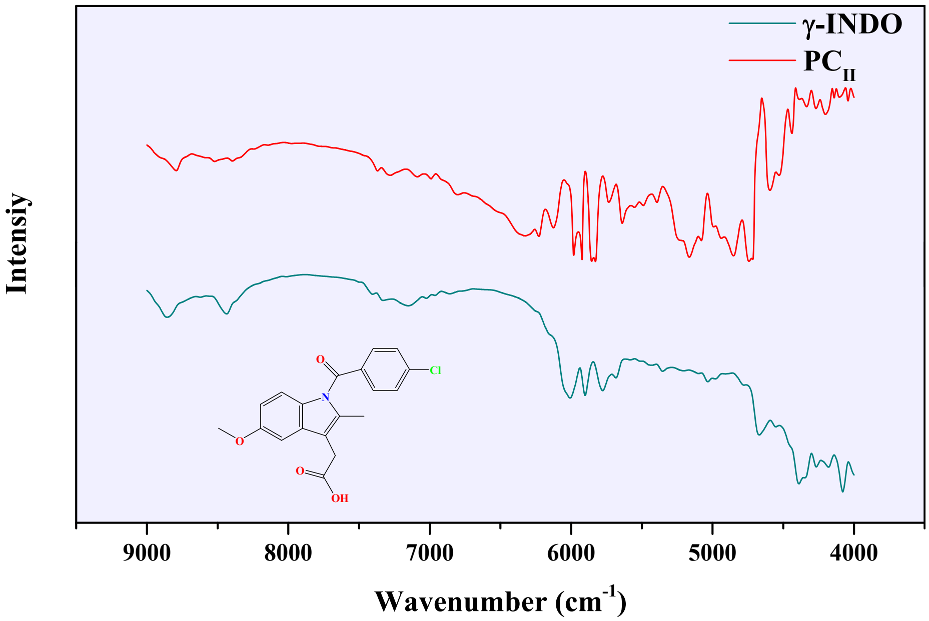

Figure. S3 Comparison between PCI loadings and reconstructed NIR spectra (after SNV+WT preprocessing) A-INDO.
